# Draft Genome Sequence of *Cyanobacterium* sp. Strain HL-69, Isolated from a Benthic Microbial Mat from a Magnesium Sulfate-Dominated Hypersaline Lake

**DOI:** 10.1128/genomeA.01583-17

**Published:** 2018-02-08

**Authors:** J. M. Mobberley, M. F. Romine, J. K. Cole, Y. Maezato, S. R. Lindemann, W. C. Nelson

**Affiliations:** a Biological Sciences Division, Pacific Northwest National Laboratory, Richland, Washington, USA

## Abstract

The complete genome sequence of *Cyanobacterium* sp. strain HL-69 consists of 3,155,247 bp and contains 2,897 predicted genes comprising a chromosome and two plasmids. The genome is consistent with a halophilic nondiazotrophic phototrophic lifestyle, and this organism is able to synthesize most B vitamins and produces several secondary metabolites.

## GENOME ANNOUNCEMENT

Cyanobacteria in phototrophic microbial mats are responsible for most of the primary production, provide fixed nitrogen and sulfur to the community, and contribute to the structural integrity of the mat (1, 2). We present here the complete genome sequence of the coccoid unicellular *Cyanobacterium* sp. strain HL-69 (CHL-69), which was derived from a microbial mat from the magnesium sulfate-dominated hypersaline Hot Lake in northern Washington (3, 4). CHL-69 was isolated from a Hot Lake mat enrichment culture by streaking until axenic on Hot Lake autotroph (HLA) medium, which is BG-11 amended to mimic Hot Lake water (5).

CHL-69 genomic DNA was extracted using a modified cetyltrimethylammonium bromide (CTAB) protocol (5) and was sequenced by the Department of Horticulture Genomics Lab at Washington State University in Pullman, WA, USA, on a PacBio RS II platform, which generated 60,773 reads with a mean length of 5,905 nucleotides (nt). *De novo* assembly with Hierarchical Genome Assembly Process (HGAP) (SMRT portal version 2.2.0) (Pacific Biosciences) (6) yielded 5 unique contigs. Gaps and sequence errors were resolved using assembled shotgun metagenome data (Illumina HiSeq) from the enrichment culture (https://github.com/jenmobberley/CyanobacteriumHL69). Gene prediction was performed with Prodigal (7) and through the Rapid Annotations using Subsystems Technology (RAST) server (8), and rRNAs and tRNAs were identified with Rfam (9). Genes were assigned functional annotation by use of information from the RAST server (8), BlastKOALA (10), and TIGRFAMs (11).

The genome of CHL-69 consists of a circular chromosome (3,155,247 bp) with an average G+C content of 37.8% and two plasmids, pCHL69-1 (86,432 bp) and pCHL69-2 (55,266 bp), with average G+C contents of 34.1% and 35.32%, respectively. Sequence analysis revealed 3,039 coding sequences, 9 rRNAs, and 44 tRNAs. The chromosome contained a putative prophage as well as a clustered regularly interspaced short palindromic repeat (CRISPR)-*cas* subtype I-D system. Each plasmid contained *parA* and toxin-antitoxin genes, which suggests that the plasmids are maintained at a low copy number. Average nucleotide identity (ANI) calculations showed that the HL-69 genome was 95.8% identical to that of the freshwater isolate *Cyanobacterium* sp. strain IPPAS B-1200 (3,410,249 bp) (GenBank accession no. LWHC00000000) (12) and 82.75% identical to that of the soda lake isolate *Cyanobacterium stanieri* PCC 7202 (3,163,381 bp) (GenBank accession no. CP003940) (13).

The nutritional dependencies of *Cyanobacterium* sp. HL-69 were revealed through metabolic reconstruction. HL-69 contains nitrate assimilation genes but lacks nitrogenase, supporting experiments showing HL-69 grows on nitrate but not dinitrogen (Y.M. and J.K.C, unpublished data). The genome of HL-69 indicates it is auxotrophic for vitamin B_12_ and is capable of salvage through an ABC transporter (*btuBFCD*). HL-69 is prototrophic for B_2_, B_6_, B_7_, and B_9_; however, the presence of genes for uptake of B_7_ (*bioY*) and B_9_ (*folT*) suggests it might be conditionally syntrophic for those vitamins (14). Consistent with CHL-69 growing under a wide range of salinity and light conditions, stress response pathways were identified, such as biosynthesis of the osmolytes glucosyl-glycerol (*ggpS*) and choline (*glpQ*), as well as the UV protectant mycosporine, which may be induced by oxidative stress due to high light levels (15).

### Accession number(s).

This whole-genome shotgun project has been deposited in GenBank under the accession no. CP024912 (CHL-69), CP024913 (pCHL69-1), and CP024914 (pCHL69-2). The versions described in this paper are the first versions, CP024912.1, CP024913.1, and CP024914.1. The metagenome for the cyanobacterial enrichment culture is publically accessible in JGI’s Integrated Microbial Genomes and Microbiomes (IMG) under IMG Genome ID 3300005412.

## References

[1] Wong HL, Ahmed-Cox A, Burns BP 2016 Molecular ecology of hypersaline microbial mats: current insights and new directions. Microorganisms 4:6. doi:10.3390/microorganisms4010006. PMC5029511 27681900

[2] Mobberley JM, Lindemann SR, Bernstein HC, Moran JJ, Renslow RS, Babauta J, Hu D, Beyenal H, Nelson WC 2017 Organismal and spatial partitioning of energy and macronutrient transformations within a hypersaline mat. FEMS Microbiol Ecol 93. doi:10.1093/femsec/fix028. PMC5812542 28334407

[3] Lindemann SR, Moran JJ, Stegen JC, Renslow RS, Hutchison JR, Cole JK, Dohnalkova AC, Tremblay J, Singh K, Malfatti SA, Chen F, Tringe SG, Beyenal H, Fredrickson JK 2013 The epsomitic phototrophic microbial mat of Hot Lake, Washington: community structural responses to seasonal cycling. Front Microbiol 4:323. doi:10.3389/fmicb.2013.00323. 24312082 PMC3826063

[4] Zachara JM, Moran JJ, Resch CT, Lindemann SR, Felmy AR, Bowden ME, Cory AB, Fredrickson JK 2016 Geo- and biogeochemical processes in a heliothermal hypersaline lake. Geochim Cosmochim Acta 181:144–163. doi:10.1016/j.gca.2016.02.001.

[5] Cole JK, Hutchison JR, Renslow RS, Kim Y-M, Chrisler WB, Engelmann HE, Dohnalkova AC, Hu D, Metz TO, Fredrickson JK, Lindemann SR 2014 Phototrophic biofilm assembly in microbial-mat-derived unicyanobacterial consortia: model systems for the study of autotroph-heterotroph interactions. Front Microbiol 5. doi:10.3389/fmicb.2014.00109. PMC3985010 24778628

[6] Chin CS, Alexander DH, Marks P, Klammer AA, Drake J, Heiner C, Clum A, Copeland A, Huddleston J, Eichler EE, Turner SW, Korlach J 2013 Nonhybrid, finished microbial genome assemblies from long-read SMRT sequencing data. Nat Methods 10:563–569. doi:10.1038/nmeth.2474. 23644548

[7] Hyatt D, Chen GL, LoCascio PF, Land ML, Larimer FW, Hauser LJ 2010 Prodigal: prokaryotic gene recognition and translation initiation site identification. BMC Bioinformatics 11:119. doi:10.1186/1471-2105-11-119. 20211023 PMC2848648

[8] Aziz RK, Bartels D, Best AA, DeJongh M, Disz T, Edwards RA, Formsma K, Gerdes S, Glass EM, Kubal M, Meyer F, Olsen GJ, Olson R, Osterman AL, Overbeek RA, McNeil LK, Paarmann D, Paczian T, Parrello B, Pusch GD, Reich C, Stevens R, Vassieva O, Vonstein V, Wilke A, Zagnitko O 2008 The RAST server: Rapid Annotations using Subsystems Technology. BMC Genomics 9:75. doi:10.1186/1471-2164-9-75. 18261238 PMC2265698

[9] Nawrocki EP, Burge SW, Bateman A, Daub J, Eberhardt RY, Eddy SR, Floden EW, Gardner PP, Jones TA, Tate J 2015 Rfam 12.0: updates to the RNA families database. Nucleic Acids Res 43:D130–D137. doi:10.1093/nar/gku1063. 25392425 PMC4383904

[10] Kanehisa M, Sato Y, Morishima K 2016 BlastKOALA and GhostKOALA: KEGG tools for functional characterization of genome and metagenome sequences. J Mol Biol 428:726–731. doi:10.1016/j.jmb.2015.11.006. 26585406

[11] Haft DH, Selengut JD, White O 2003 The TIGRFAMs database of protein families. Nucleic Acids Res 31:371–373. doi:10.1093/nar/gkg128. 12520025 PMC165575

[12] Starikov AY, Usserbaeva AA, Sinetova MA, Sarsekeyeva FK, Zayadan BK, Ustinova VV, Kupriyanova EV, Los DA, Mironov KS 2016 Draft genome sequence of *Cyanobacterium* sp. strain IPPAS B-1200 with a unique fatty acid composition. Genome Announc 4(6):e01306-16. doi:10.1128/genomeA.01306-16. PMC5114388 27856596

[13] Stanier RY, Deruelles J, Rippka R, Herdman M, Waterbury JB 1979 Generic assignments, strain histories and properties of pure cultures of cyanobacteria. Microbiology 111:1–61. doi:10.1099/00221287-111-1-1.

[14] Romine MF, Rodionov DA, Maezato Y, Osterman AL, Nelson WC 2017 Underlying mechanisms for syntrophic metabolism of essential enzyme cofactors in microbial communities. ISME J 11:1434–1446. doi:10.1038/ismej.2017.2. 28186498 PMC5437353

[15] Wada N, Sakamoto T, Matsugo S 2013 Multiple roles of photosynthetic and sunscreen pigments in cyanobacteria focusing on the oxidative stress. Metabolites 3:463–483. doi:10.3390/metabo3020463. 24958001 PMC3901267

